# Drug Repurposing: An Emerging Strategy in *Staphylococcus aureus* Infections

**DOI:** 10.4014/jmb.2507.07048

**Published:** 2025-10-29

**Authors:** Yimin Li, Pengfei She, Yiqing Liu, Shaowei Guo, Guanqing Huang, Dan Xiao, Yong Wu

**Affiliations:** 1 Department of Laboratory Medicine, The First Hospital of Changsha (The Affiliated Changsha Hospital of Xiangya School of Medicine, Central South University), 410005, Changsha, Hunan, P.R. China; 2 Department of Laboratory Medicine, Third Xiangya Hospital of Central South University, Changsha, 410013, Hunan, P.R. China

**Keywords:** Drug repurposing, *Staphylococcus aureus*, resistance, antibiotic discovery

## Abstract

Infections are a growing global threat to public health, and the rapid spread of drug‐resistant pathogens is a big challenge. The development and approval of novel antimicrobials targeting multi-drug-resistant infections lag significantly behind the rapid emergence of resistance. For these threats and challenges, new strategies are critically needed. Drug repurposing, using approved or investigational drugs for other clinical conditions, has emerged as an alternative approach for rapid identification of effective antibacterial agents. Although drug repurposing is not novel, recent years have witnessed the development of novel methodologies enabling its systematic and rational implementation. This review summarizes methodological approaches for antimicrobial repurposing against *Staphylococcus aureus*, evaluates current research progress, and highlights persistent challenges and promising prospects. Collectively, drug repurposing is poised to deliver clinically impactful antimicrobials, offering a pragmatic complement to novel drug discovery in the global fight against antimicrobial resistance.

## Introduction

The majority of antibiotics currently in clinical use were primarily developed during the 1960s and 1970s through phenotypic screening of natural products and semi-synthetic derivatives [1]. However, the widespread use of these antimicrobial agents, combined with insufficient infection control practices, has driven the rapid evolution of drug-resistant bacterial strains [2]. *Staphylococcus aureus*, a pathogen distinguished by its diverse virulence factors, poses significant challenges to modern antimicrobial therapy. Its capacity to develop resistance mechanisms against multiple classes of clinically important antibiotics has resulted in a progressively worsening antimicrobial resistance crisis. Of particular concern is methicillin-resistant *S. aureus* (MRSA), a leading causative agent of both healthcare-associated and community-acquired infections worldwide. Clinical infections caused by MRSA are associated with elevated morbidity and mortality rates, constituting a significant burden on global public health systems. Common clinical presentations include bacteremia, respiratory tract infections, skin and soft tissue infections, osteomyelitis, and septic arthritis. Although vancomycin remains the primary therapeutic option for MRSA treatment, the emergence of *S. aureus* strains with diminished vancomycin susceptibility has created new obstacles in clinical management [3]. These findings collectively indicate a global escalation of antibiotic resistance in *S. aureus*, underscoring the urgent need for novel therapeutic interventions and antimicrobial development.

Advances in molecular biology and bacterial genome analysis have made target-based high-throughput drug screening the primary strategy for antimicrobial development in recent decades [4, 5]. However, very few candidate compounds have progressed to clinical trials. Additionally, pharmaceutical companies show limited interest in antimicrobial research due to low profitability and short antibiotic market viability [6]. This shortage of effective antibiotics requires urgent action from both medical professionals and the public to resolve this critical health challenge.

Drug repurposing, which entails identifying novel therapeutic applications for approved or investigational drugs beyond their original medical indications. is an effective strategy to address bacterial resistance [7]. Approximately 20–30 new drugs receive FDA approval annually, with about 30% being repurposed agents [8]. The classic cases of drug repurposing include thalidomide, sildenafil, raloxifene, minoxidil and so on. This strategy has been successfully applied in developing antibacterial, anti-cancer [9], anti-viral drugs [10], anti-inflammatory [11], and anti-parasitic [12] agents. Compared to traditional drug development, this approach offers advantages including improved antibacterial efficacy, reduced single-drug dosage, fewer side effects, prevention of resistance development, lower costs, and faster clinical validation [13, 14]. Another type of drug repurposing is different from directly applying approved or investigational drugs for a new indication, compounds identified as potential hits need undergo further optimization. Although this lead optimization process is more complex, necessitating full clinical development for the newly generated compounds, it presents a significant advantage: the potential to eliminate undesirable side-effects or off-target interactions associated with the compound's original use.

Given the challenges of pathogenic bacteria, researchers should prioritize increased investment in drug repurposing to develop new therapies for infections caused by multidrug-resistant pathogens like *S. aureus*. To support this effort, this review systematically analyzes methodological approaches for antimicrobial repurposing against *S. aureus*, evaluating current research progress while highlighting persistent challenges and promising prospects.

## Approaches to Antibacterial Drug Repurposing

**Fig. 1:**
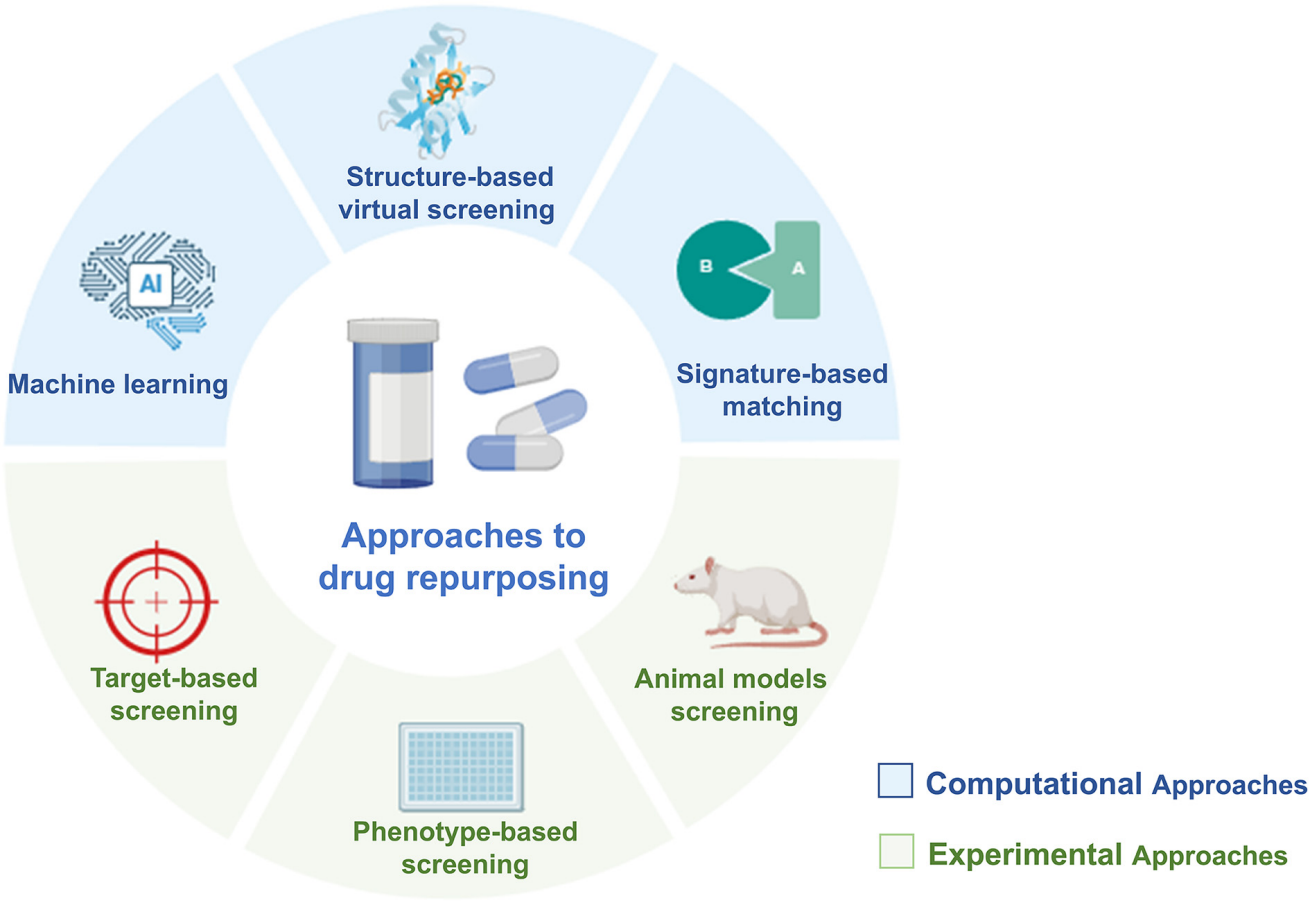
Approaches to drug repurposing. Drug repurposing assays are generally classified into two categories: computational and experimental approaches. Combining both methods often produces optimal repurposing outcomes.The figure has been designed using resources from biorender.

Antibacterial drug repurposing strategies are broadly categorized into two main categories: computational and experimental approaches. Combining both methods often produces optimal repurposing outcomes Fig. 1.

### Computational Approaches

Computational approaches using machine learning (ML) can effectively analyze biological data patterns. Systematic analysis of multi-omics data (genomics, transcriptomics, proteomics, metabolomics), chemical structures, molecular docking results, and clinical records provides new insights for antibacterial drug repurposing.

**ML-Based methods.** ML models integrate molecular fingerprints, docking results, and multi-omics datasets to predict repurposing drugs. Deep neural networks optimized via ensemble learning identified SU3327, a c-Jun *N*-terminal kinase inhibitor, as effective against *Escherichia coli* across >10^7^ million screened molecules [15]. Algorithms combining FP2 molecular fingerprints with Random Forest/support vector machine/multi-layer perception identified eight novel structures candidates, suggesting unexplored antibacterial pathways [16]. Despite progress, ML efficacy relies on multi-source data integration, necessitating adaptive algorithms for rapid pathogen response.

**Structure-based virtual screening.** Structure-based virtual screening is a computational approach commonly applied in the early phases of drug discovery to identify molecules with high target affinity and specificity. Fenoprofen, a nonsteroidal anti-inflammatory drug (NSAID), was repurposed as a SaeR inhibitor in *S. aureus* via virtual screening [17]. Combined docking, MD simulations, and QM/MM calculations revealed Lumacaftor’s strong binding to *Staphylococcus* FemX enzyme, highlighting therapeutic potential [18]. Screening 4,500 FDA-approved ligands against mutated *S. aureus* IleS identified buclizine and emetine as lead candidates [19].

**Signature-based matching.** Signature matching is a computational method that compares a drug's characteristic profiles with pharmacological agents or clinical phenotypes through multi-omics datasets, adverse event reports, and structural chemistry analyses [13, 20]. Researchers integrated *S. aureus* endophthalmitis transcriptomics with connectivity map analysis, predicting clofilium tosylate and glybenclamide as antimicrobial agents that reverse infection-associated gene signatures [21, 22].

### Experimental Approaches

Experimental approache assays can be generally divided into (i) target-based screening, (ii) phenotype-based screening, and (iii) animal models.

**Target-based screening.** This methodology focuses on bacterial targets including cell wall biosynthesis, protein synthesis, nucleic acid metabolism, membrane stability, essential metabolic pathways, and bacterial-specific enzymes. Widely adopted in drug repurposing studies due to its operational simplicity [23, 24], this approach enables mechanism-driven discovery with clear therapeutic rationale. Nevertheless, it faces limitations including target dependency, challenges in novel target identification, high false-positive rates, and narrowed therapeutic scope that may overlook broader opportunities and accelerate resistance development.

**Phenotype-based screening.** This strategy evaluates compound effects through direct observation of bacterial growth inhibition and viability changes [25, 26]. In contrast to target-based methods, it does not require prior knowledge of specific molecular targets and eliminate target hypothesis-driven screening bias. While particularly effective for identifying multi-target agents and adaptable to high-throughput formats, this approach presents challenges in lead optimization due to unclear mechanisms of action, potential off-target effects, and subsequent requirement for extensive mechanistic studies.

**Animal models for antibacterial drug repurposing.** Animal models constitute indispensable tools for the preclinical evaluation of novel antimicrobial compounds. Murine models (mice and rats) are commonly utilized owing to their physiological parallels with humans, facilitating the assessment of drug efficacy, pharmacokinetics, toxicity profiles, and antimicrobial resistance development. Alternative models such as zebrafish, *Caenorhabditis elegans*, and fruit flies enable rapid and cost-effective screening [27-30]. Within *in vivo* infection studies, the primary metric centers on host survival rather than direct bacterial killing. However, these models present limitations including substantial costs and time requirements, ethical considerations, interspecies variations potentially confounding drug response predictions, and challenges associated with experimental reproducibility and clinical translatability.

## Drug Repurposing Candidates for Anti-*S. aureus*

Drug candidates for antimicrobial repurposing generally fall into two categories; (i) direct antibacterial agents exhibiting intrinsic bactericidal/bacteriostatic activity against planktonic or biofilm-embedded pathogens, and (ii) antimicrobial adjuvants including host-directed therapies, ant virulence drugs, anti-resistance drugs. This systematic evaluation examines both paradigms, analyzing their molecular targets, spectrum of activity, and clinical translation potential. And Table S1 systematically characterizes the drug repurposing candidates, listing their original therapeutic class, approved mechanisms of action, proposed antimicrobial mechanisms against *S. aureus*, minimum inhibitory concentrations (MICs), and approaches used in drug repurposing.

### Direct Antibacterial Agents

Direct antibacterial agents are classified and analyzed based on their regulatory-approved clinical indications

**Anti-inflammatory drugs.** Auranofin (AUR), a clinically used gold-containing compound approved by the FDA for rheumatoid arthritis, demonstrates antibacterial activity against multiple *S. aureus* strains with MICs ranging from 0.0625 to 0.5 μg/ml [31-41]. The *in vivo* antibacterial efficacy of AUR has been confirmed through multiple animal models [31-33]. AUR exhibited its antibacterial activity by inhibiting thioredoxin reductase, which disrupts the bacterial redox balance and increases cellular sensitivity to oxidative stress [34]. Moreover, it interacts with selenium to impair selenoprotein biosynthesis by targeting the C-terminal region of Sec residues, further destabilizing cellular redox equilibrium [36]. Thioredoxin reductase was not the sole target of AUR in *S. aureus*. AUR also influenced key biological processes, including cell wall synthesis, DNA replication, and bacterial protein synthesis [37]. Synergistic interactions have been observed between AUR and antibiotics including linezolid, fosfomycin, ciprofloxacin, or phenethyl isothiocyanate [32, 37, 38]. AUR structural derivatives such as MH05 and AUR-coated catheters have also shown high potency against *S. aureus* [31, 39, 40]. The chemical modification of AUR represents a promising strategy for enhancing its antibacterial properties.

Ibuprofen is a NSAID. Previous studies have demonstrated its inhibitory effects against multiple bacterial species, including *Streptococcus pneumoniae*, *Candida albicans*, *E. coli* and *S. aureus* [42-45]. In the study of Oliveira *et al*., the MICs of ibuprofen against standard and resistant *S. aureus* strains ranged from 500 to 1,600 mg/l. This antibacterial effect was linked to cytoplasmic membrane destabilization, causing potassium ion efflux and altered surface properties (reduced hydrophobicity and modified surface tension). Ibuprofen also showed potent antibiofilm activity through metabolic inactivation and colony-forming unit (CFU) reduction, especially against adherent and mature biofilms [46]. However, clinical application requires caution, as the MICs (500-1,600 mg/l) presented in this study were higher than the maximum plasma concentration (100 mg/l). Commercially available topical formulations contain 5% (w/w) ibuprofen, which exceeds the antimicrobial-effective concentrations identified in this study. Consequently, ibuprofen can initially serve as an antiseptic or disinfectant in topical formulations.

Diclofenac (DC), one of the most widely used NSAID, displays antibacterial activity against *S. aureus* [47-49]. The study of Zhang *et al*. focus on the synergistic effects of DC and β‐lactams. DC combined with oxacillin could inhibit biofilm formation and reduce biofilm biomass both *in vivo* and *in vitro*. Proteomic and transcriptomic analyses showed DC-mediated downregulation of: Cell wall biosynthesis genes (*murA*, *murC*, *femA*), Biofilm-related genes (*altE*, *fnbP*) and β-lactam resistance genes (*mecA*, *mecR*, *blaZ*, *femB*). Abbas *et al*. and Elmesseri *et al*. further reported DC's anti-virulence effects, suppressing staphyloxanthin (STX) production critical for MRSA immune evasion, while reducing expression of STX synthesis genes, regulator *sigB*, and virulence factors (*e.g.*, *hla*).

Meloxicam (MXM) demonstrated potent antibacterial efficacy against MRSA by significantly inhibiting STX production (80.6–96.7% inhibition at MXM 59 μg/ml) [49]. This inhibition correlated with downregulation of key virulence genes (*crtM*, *crtN*, *sigB*, *hla*) involved in STX synthesis and α-hemolysin production. And in the study of Altaf *et al*., MXM showed partial synergism with oxytetracycline and gentamicin against MRSA, enhancing antibacterial activity and potentially reversing resistance *in vivo* and *in vitro* [50].

Celecoxib, a selective cyclooxygenase-2 (COX-2) inhibitor, exhibits antibacterial activity against *S. aureus*, including MRSA strains, with MICs ranging from 32 to 64 μg/ml [51]. It potentiates the effect of antibiotics like ampicillin by altering the membrane potential and permeability of *S. aureus*, specifically affecting the Na^+^/K^+^ ion transporter, thereby increasing the uptake of ampicillin and enhancing its antibacterial effect [52]. Celecoxib derivatives Cpd36/Cpd46 exhibited antimicrobial activity against MRSA and related *Staphylococci* by targeting YidC2 translocase—a membrane protein essential for bacterial membrane protein assembly [53]. This inhibition identified the potential of YidC2 as a novel therapeutic target.

Ebselen, an organoselenium compound with broad antioxidant properties, exhibited significant antibacterial effects against MDR *S. aureus* in topical treatment [54]. Ebselen targeted the bacterial thioredoxin reductase (TrxR), effectively disrupting the bacteria's redox balance, elevating reactive oxygen species (ROS) levels, suppressing growth, and compromising membrane integrity. In the skin wound infection rat model, ebselen significantly reduced the bacterial load in skin infection sites, decreased the expression of inflammatory cytokines, and promoted wound healing. Additionally, the combination of ebselen with curcumin exhibited synergistic antibacterial effects against *S. aureus*. Saha *et al*. developed inhalable ebselen dry powder formulations showing potent anti-*S. aureus* activity for treating respiratory tract infections [55].

**Anti-parasitic agents.** Folliero *et al*. screened avermectin-class anti-parasitic agents and identified selamectin as a potent antibacterial agent of *S. aureus* strains [56]. The Mean MIC_90_ values against to tested *S. aureus* strains (clinical isolates with different antibiotic resistance phenotypes) were 6.2 μg/ml. Scanning electron microscopy (SEM) analysis revealed that selamectin caused relevant cell surface damage. Selamectin significantly reduced biofilm biomass in a dose-dependent manner, exhibiting a minimum biofilm eradication concentration for 50%of strains (MBEC_50_) of 5.89 μg/ml. At MIC concentrations, selamectin reduced intracellular bacterial loads by 81.3% without hemolytic or cytotoxic effects. Conversely, Lim *et al*. did not report the antibacterial activity of selamectin up to 256 μg/ml against *S. aureus*. The variation can be attributed to the growth conditions, divergence in strains, and methodologies employed [57].

The salicylanilide family of anthelmintic drugs, oxyclozanide and niclosamide demonstrated significant *in vivo* and *in vitro* efficacy against MRSA [58, 59]. Oxyclozanide could kill *S. aureus* clinical isolates resistant to methicillin, vancomycin, linezolid, or daptomycin revealed MICs ranging from 0.125 to 1 μg/ml [58]. Mechanistic studies demonstrated that oxyclozanide disrupted bacterial membranes and stimulated ROS overproduction. Furthermore, oxyclozanide emerged as a novel antibacterial adjuvant that markedly enhances the effectiveness of tetracycline antibiotics by increasing intracellular tetracycline accumulation in bacteria. Additionally, it diminished virulence protein production in *S. aureus* and neutralizes α-hemolysin activity, thereby reducing bacteria-induced inflammatory responses. The MICs of niclosamide were 0.06-0.125 μg/ml against *S. aureus*, showing excellent antibacterial activity *in vitro* [59]. Mechanism experiments revealed that niclosamide altered the surface morphology of *S. aureus*, reduced its intracellular ATP levels in a dose-dependent manner, and inhibited its haemolytic activity through expression regulation.

Cheng *et al*. conducted high-throughput screening for bi-functional transglycosylases (TGase) inhibitors (a peptidoglycan synthesis-associated protein), identifying salicylanilide analogues with an additional aryl group that exhibited strong TGase inhibition activities. Pauk *et al*. [60] and Kratky *et al*. [61, 62] documented salicylanilide derivatives with antibacterial activities against various bacteria species including MRSA.

Triclabendazole (TCBZ), originally developed to treat Fasciola hepatica, has shown antibacterial activity against Gram-negative bacteria, including *Klebsiella pneumoniae*, *Pseudomonas aeruginosa*, *Acinetobacter baumannii* and *E. coli* in combination with polymyxin B, as well as against Gram-positive bacteria such as MRSA, Methicillin-Susceptible *S. aureus* (MSSA), Vancomycin-Resistant *Enterococcus* (VRE) and *S. pneumoniae* [63]. TCBZ’s safety and efficacy was also evaluated as a bioluminescent mouse model of *S. aureus* sepsis. But it couldn’t eliminate infection with selected dosage regimen. Further pharmacokinetics, dosing optimisation and mechanism studies of TCBZ still should be identified.

Tafenoquine (TAF), an antimalarial agent, exhibited potent antibacterial activity against MRSA, including its resistant phenotypes such as biofilms and persister cells. The research highlighted TAF's novel application in combating MRSA through selective bacterial cell membrane disruption without inducing resistance, supported by *in vitro* and *in vivo* evidence of its safety and efficacy [64].

Pearce *et al*. examined a primaquine-polyamine hybrid, which was initially synthesized as a possible anti-malarial candidate, and conducted a series succinylprimaquine-polyamine (SPQ-PA) conjugates [65]. SPQ-PA3-10-3 and SPQ-PA3-8-3 exhibited moderate intrinsic antibacterial activity against *S. aureus*. Specifically, SPQ-PA3-10-3 demonstrated a MIC of 3.3 μM, while SPQ-PA3-8-3 exhibited a MIC of 13.7 μM. The antibacterial activity of SPQ-PA conjugates against *S. aureus* is influenced by various factors, including the substituents on the quinoline ring, the length and position of the ethylenediamine chain, and the substituents on the polyamine core. SAR studies reveal that the 6-methoxy substituent, a 10-carbon ethylenediamine chain, and an 8-substituted quinoline ring are crucial for activity. Further research is still needed to develop SPQ-PA conjugates with higher activity and selectivity.

Repurposing study identified the clinically approved anthelmintic drug bithionol as an effective antibiotic against MRSA persisters [66]. Bithionol effectively inhibited MRSA persister cells by damaging Gram-positive bacterial membranes with high specificity over mammalian cell membranes. Its efficacy depends on lipid binding affinity, penetration depth, and molecular size. The effective elimination of MRSA persister cells necessitates membrane-targeting compounds to provoke critical-level membrane perturbations. This mechanistically linked phenomenon positions membrane fluidification as a biophysical indicator to identify and measure potent antipersister compounds. When combined with gentamicin, bithionol significantly reduced MRSA in a mouse thigh infection model.

Nitazoxanide inhibited *S. aureus* growth (MIC = 16 μg/ml) and synergistically enhanced linezolid efficacy *in vitro* and *in vivo* [67].

**Antipsychotics.** Penfluridol (PF), a long-acting antipsychotic, demonstrated concentration-dependent antibacterial activity against *S. aureus* (MIC = 4–8 μg/ml; MBC = 16–32 μg/ml) [68]. PF dose-dependently reduced both *S. aureus* biofilm and persister cells in a dose-dependent manner. The underlying antibacterial mechanism may be membrane disruption and ATP release caused by PF. PF showed both antimicrobial and anti-inflammatory activity in different mouse model including skin wound infection,subcutaneous abscess, and acute peritonitis [68]. PF demonstrated little hemolytic activity on human erythrocytes and limited cytotoxicity.

Fluoxetine (FLX) is a selective serotonin reuptake inhibitor. Sousa *et al*. investigated that FLX presented antibacterial effects against *S. aureus*, *P. aeruginosa* and *E. coli* for both standard and multidrug resistant strains. Meanwhile, FLX presented synergistic effects with gentamicin and erythromycin [69]. Neto *et al*. further observed FLX damaged MRSA membrane, induced DNA fragmentation and caused phosphotidylserine exposure in the cytoplasmic membrane [70]. Some studies developed different FLX delivery systems to improve its antibacterial ability. Drug delivery optimization studies have engineered FLX-loaded starch nanocapsules (MIC = 95–190 μg/ml) and galactomannan microparticles (MIC = 127.5–255 μg/ml) [71, 72].

Nehme *et al*. assessed antipsychotics' antibacterial effects: Atypical agents, reserpine, haloperidol, and sulpiride showed no activity (MIC >1,024 μg/ml). Phenothiazines and thioxanthenes exhibited strain-dependent MICs (32–64 μg/ml against Gram-positive *S. aureus*; 64–128 μg/ml against Gram-negative pathogens), with structural parallels (*e.g.*, double bond substitution) explaining potency similarities. Gram-negative resistance arose from outer membrane barriers (LPS gel-layer, restricted porins, efflux pumps), notably in *P. aeruginosa* (MIC 256–1,024 μg/ml). Subsequently, they prepared lipid nanocapsules (LNCs) to enhance the antibacterial efficiency of phenothiazines and thioxanthenes [73].

Thioridazine (TZ)'s exhibited activity *in vitro* against *S. aureus* [74]. Costa *et al*. found TZ significantly lowers ciprofloxacin MIC in efflux-pump resistant *S. aureus*, suggesting its role as an efflux inhibitor [75]. Photoproducts of TZ by laser irradiation were particularly effective against methicillin- and ciprofloxacin-resistant *S. aureus*. Molecular docking studies indicated that these photoproducts potentially exerted inhibitory effect primarily by inhibiting the activities of penicillin-binding proteins 3 (PBP3) and 2a (PBP2a) [76]. The toxicity of individual photoproducts formed during TZ irradiation remains unpredictable, necessitating *in vitro* and *in vivo* safety studies.

**Anticancer drugs.** Curcumin is an anticancer drug and exhibited potent antimicrobial activity against *S. aureus* and *P. aeruginosa* (MICs 100 μg/ml and 50μg/ml, respectively) in the study of Kamurai *et al* [77]. They found curcumin could also enhance the antibacterial activity of ciprofloxacin on both two bacteria and curcumin might work by damaging the bacterial membrane. In the other study of Mun *et al*., curcumin had a remarkable antibacterial effect against *S. aureus* with permeability enhancers and ATPase inhibitors. Meanwhile curcumin could reduce PBP2a levels in MRSA [78].

Tyrosine kinase inhibitors (TKIs) represent a class of targeted therapeutic agents characterized by their capacity to selectively inhibit aberrant kinase signaling pathways. Ceritinib is a TKIs of anaplastic lymphoma kinase and used to treat non-small cell lung cancer. Liu *et al*. discovered the antimicrobial efficacy of ceritinib against *S. aureus* planktonic cells, persisters and biofilms *in vitro* [79]. Ceritinib also ameliorates *S. aureus* infection in subcutaneous abscesses mouse model. Through mechanisms research, ceritinib possibly kills bacteria by breaking their membranes and activating ROS. Crizotinib, a multi-targeted TKI, effectively reduced bacterial ATP production by inhibiting the CTP synthase PyrG. This inhibition disrupted pyrimidine metabolism, thereby impairing DNA synthesis [80]. Notably, crizotinib demonstrated lower propensity for resistance development compared to ampicillin. Crizotinib also exhibited synergistic antibacterial effects with clindamycin and gentamicin, enhanced survival and mitigated inflammation in the mouse pneumonia model.

Research have focused on the identification of small molecule kinase inhibitors with activity against *S. aureus* and confirmed sorafenib as a potent hits [25]. Following the synthesis of 72 sorafenib scaffold analogues, subsequent identified PK150 as a promising lead compound. PK150 exhibited a 10-fold enhancement in growth inhibition against *S. aureus*, demonstrated efficacy against bacterial persister cells, effectively suppressed biofilm formation, and demonstrated *in vivo* efficacy in neutropenic mouse thigh model. Chemical proteomics further identified that suppression of menaquinone biosynthesis coupled with impaired protein secretory pathways constitute probable mechanisms of action.

Ethyl bromopyruvate (EBP) is a derivative of bromopyruvate (an anticancer agent that targets the Warburg effect). Kumar *et al*. demonstrated that EBP exhibits broad-spectrum antimicrobial activity, showing comparable potency against both drug-sensitive and multidrug-resistant strains of Mycobacterium tuberculosis and ESKAPE pathogens (MIC range: 32-64 mg/l). It showed concentration-dependent bactericidal activity, reducing bacterial counts by 9log_10_ cfu/mL in 24 h, similar to vancomycin. It was particularly effective in inhibiting S.aureus biofilm formation, surpassing levofloxacin and matching vancomycin. In a murine thigh infection model, EBP significantly reduced bacterial counts, showing efficacy comparable to vancomycin at 1/25th of the dose [81].

Etoposide, a topoisomerase II inhibitor used in testicular/lung cancers, exhibits anti-*S. aureus* activity (MICs 64-256 μg/ml) by stabilizing DNA-topoisomerase cleavage complexes and blocking re-ligation, disrupting bacterial DNA replication [82]. Etoposide interacted with the conserved TOPRIM domain of the GyrB subunit in bacterial DNA gyrase, a mechanism that was similar to but distinct from that of fluoroquinolones, which allowed it to overcome fluoroquinolone resistance. Ganesan *et al*. combined etoposide with eggshell-derived hydroxyapatite and this novel antibacterial materialresults exhibited significant antibacterial activity against *S. aureus*, effectively inhibiting bacterial growth and reducing biofilm formation [83].

She *et al*. investigated the antimicrobial potential of the anticancer drug idarubicin against MRSA. The experimental findings revealed that idarubicin exerted antimicrobial effects by disrupting bacterial cell membranes and interfering with DNA topoisomerase IIA subunits, which led to significant activity against both planktonic MRSA cells and biofilms. A notable highlight was the synergistic effect observed between idarubicin and fosfomycin, which enhanced idarubicin’s antimicrobial potency while also reducing its cytotoxicity and cardiotoxicity [84].

Gemcitabine (GEM), a nucleoside analog clinically approved for ovarian cancer and non-small cell lung carcinoma, along with its lipophilic derivative CP-4126, demonstrated potent antibacterial efficacy against clinically relevant multidrug-resistant *S. aureus* strains, including MSSA, MRSA, and glycopeptide-intermediate *Staphylococcus aureus*(GISA).The study identified the potential role of the deoxyribonucleoside kinase gene *SadAK* in the antibacterial activity of GEM[85]. GEM showed synergistic effects when combined with gentamicin and cefoxitin [85, 86]. Among the GEM prodrugs, cycloSal derivatives exhibited optimal antibacterial activity against Gram-positive bacteria, though less potent than GEM itself, with compound 20a being the most active [87]. Nucleoside analogues may combat multidrug-resistant bacterial infections despite toxicity concerns, requiring preclinical validation and analog development to reduce toxicity and resistance. Microbiota evolution in treated patients warrants further study.

5-Fluorouracil (5-FU) has been licensed for treating various common and aggressive cancers. Cohen *et al*. revealed 5-FU could inhibit the growth of *S. aureus* and induce cellular death [88]. Ueda *et al*. established that 5-FU effectively suppresses the formation of *S. aureus* biofilms [89]. Zimmermann *et al*. discovered that 5-FU inhibits both DNA synthesis and protein synthesis in *S. aureus*[90]. Sedlmayer *et al*. Found that 5-FU displayed quorum-quenching properties and effectively inhibited AI-2 production and release by MRSA, *S. epidermidis*, *E. coli*, and *Vibrio harveyi* [91]. However, because 5-FU is a BCS class III drug, its low permeability restricts drug delivery. Patil *et al*. synthesized and identified that 5-FU derivatives with trihexylphosphonium substituents (6a, 6b, and 6c) exhibited significant activity against both Gram-positive and Gram-negative bacteria [92]. Capecitabine, a prodrug of 5-FU, has demonstrated anti-*S. aureus* effect both *in vitro* and *in vivo* [93, 94]. However, the antimicrobial mechanism of capecitabine remained unknown, and its pharmacokinetic properties exhibited certain limitations.

**Antifungal agents.** A topical antifungal agent oxiconazole was demonstrated antibacterial activity against *S. aureus* and *Enterococcus* spp. with MICs 2-4 μg/ml and 1-16 μg/ml. Oxiconazole could eradicate preformed *S. aureus* biofilms and showed a low propensity for resistance [95]. Oxiconazole also strongly synergized with gentamicin both *in vivo* and *in vitro*, which offered a promising combination group for mixed bacterial and fungal superficial skin infections treat.

Wang *et al*. revealed that salifungin was a promising novel antibiotic against MRSA and reduced the MICs of traditional antibiotics. Salifungin achieved its antimicrobial effects by disrupting bacterial membrane integrity, inhibiting phosphatidic acid biosynthesis, and interfering with biofilm formation and maturation. Additionally, salifungin demonstrated efficacy in wax moth and mouse infection models, exhibiting anti-inflammatory properties. With low hemolytic and cytotoxic activities, salifungin held potential for development as a therapeutic agent for human use [96].

Thiram (THM) demonstrated bacteriostatic activity against *S. aureus*, encompassing MRSA and VRSA strains. The MICs for MRSA and other resistant variants were observed to range from 2 to 8 μg/ml [97]. Specifically, the MIC_50_ and MIC_90_ values for vancomycin-susceptible, intermediate, and resistant *S. aureus* were within the range of 2-4 μg/ml and 4 μg/ml, respectively. The antibacterial efficacy of THM was found to be both time- and dose-dependent, and its activity was modulated by factors such as inoculum size, the composition of the culture medium, and environmental conditions. It is noteworthy that THM exhibited no activity against Gram-negative bacteria and presented significant toxicity, which could limit its potential systemic application.

**Antiviral drugs.** Li *et al*. [98] identified that simeprevir (SIM), an anti-hepatitis C virus drug, exhibits significant antimicrobial potential against MRSA, showing potent antibacterial activity (MIC: 2-8 μg/ml) with minimal development of *in vitro* resistance. SIM effectively inhibited *S. aureus* biofilm formation and exhibited low toxicity in both *in vitro* and *in vivo* assays. When combined with gentamicin, SIM showed synergistic antimicrobial effects, markedly reducing bacterial load in an MRSA-infected mouse model. Mechanistic studies suggest that SIM disrupts bacterial cell membranes and depletes intracellular ATP levels. Additionally, SIM restored the anti-*S. aureus* activity of polymyxins, particularly polymyxin E both *in vitro* and *in vivo* [99]. The combination of SIM with polymyxins demonstrated effective bactericidal activity against highly resistant phenotypes, including intracellular bacteria, persister cells, and biofilms, while retaining low cytotoxicity and *in vivo* toxicity, indicating its potential for therapeutic use.

Elvitegravir had demonstrated antibacterial activity against *S. aureus*, including MRSA, *in vitro* with a MIC of 4 μg/ml [100].

**Others.** Eltrombopag (EP), a non-peptide thrombopoietin receptor agonist initially licensed for the treatment of thrombocytopenia. In two studies of Lee and She, EP displayed the greatest efficacy against *S. aureus* [101, 102]. Lee *et al*. found that EP inhibited *S. aureus* growth both in cell line and mouse infection model and the antibacterial mechanism of EP is related to the activation of LytE and YokF and the gene expression in YdeL, WalR and Spx regulons. They also identified the antibacterial activity of a Class III antiarrhythmic drug dronedarone HCl and an anticancer drug ceritinib with MIC_50_ 7.6 ± 0.7 mg/l and 23.7 ± 5.3 mg/l against *S. aureus*. In the study of She *et al*., EP inhibited *S. aureus* planktonic cells, biofilm formation and eradicated the 24 h mature biofilm. EP also decreased the bacterial burden in various *in vivo* infection models. Finally, they found that EP may disrupted the protonmotive force to anti-*S. aureus*. Both studies substantiate the potential of EP as a therapeutic candidate against MRSA, while providing complementary antibacterial mechanistic.

Cinacalcet, used to treat parathyroid cancer and hyperparathyroidism, was found kills *S. aureus* by destroying bacterial membrane, respiratory metabolism and ROS production with low resistance growth [103]. Cinacalcet inhibited biofilm formation through down regulated IcaADBC by targeting IcaR. Cinacalcet’s activity *in vivo* was also observed in a pneumonia model and a biofilm infection model. Through biolayer interferometry analysis and electrophoretic mobility shift assay, Fang *et al*. believed that as an inhibitor of ica transcription, IcaR is likely to be a more attractive target for the development of antibiotics to inhibit the biofilm of antibiotic-resistant bacteria.

Disulfiram (DSF) is typically used as to treat alcoholism [104, 105]. Owing to its electrophilic nature, DSF can form disulfide bonds with biomolecules that contain cysteine residues. This interaction covalently modifies bacterial enzymes and cofactors containing thiol groups, leading to enzyme inhibition and disruption of essential cellular processes [106]. The MIC ranges were 8 to 32 μg/ml for DSF against oxacillin sodium -, linezolid-, vancomycin-resistant variants, MSSA and MRSA [97, 107, 108]. Some studies noted that DSF combined with vancomycin exhibiting synergy against vancomycin-resistant species [97, 108, 109]. Similarly, DSF and its S-octyl analog enhance the susceptibility of *S. aureus* to fosfomycin [110]. DSF holds potential as a retooled therapeutic for infections, yet its safety concerns are noteworthy. The adverse reactions spans from trivial to severe, with lower doses conveying less risk. Given the significant variability in disulfiram's pharmacokinetics among individuals, its application in humans requires prudence.

The study of Kim *et al*. presented the discovery of synthetic retinoids CD437 and CD1530, which exhibited potent bactericidal activity against MRSA, including persister cells, by disrupting lipid bilayers [111]. These compounds showed rapid bacterial eradication, synergistic effects with gentamicin, and a low potential for resistance development. Molecular dynamics simulations corroborated their membrane-disrupting action, and *in vivo* tests confirmed their efficacy in a chronic MRSA infection mouse model. Notably, a modified version of CD437, referred to as analog 2, maintained antimicrobial potency while reducing toxicity to human cells. In another study by Cheng *et al*., the adamantane moiety was identified as crucial for the antibacterial efficacy of CD437 and its analogs, with some demonstrating selectivity for cancer cells over MRSA [112]. CD5789 (trifarotene) is also a fourth-generation retinoid for the topical acne vulgaris regimen [113]. It showed strong antibacterial ability against *S. aureus* type strains and clinical isolates with different resistant patterns with MICs and MBCs 2–4 μg/ml and 4–16 μg/ml, respectively. CD5789 displayed enhanced bactericidal effects against high resistant phonotypes (including persister cells and biofilm) and synergistic antimicrobial effects with gentamycin. CD5789 is usually made into cream to treat facial and truncal acne, so it has good potential for *S. aureus* skin and soft tissue infections treatment. Unfortunately, researchers did not conduct *in vivo* experiments.

Some researchers have synthesized retinoid drug analogs. Haney *et al*. synthesized an azaborine retinoid isostere. Despite increased water solubility, the azaborine analog showed lower antibacterial activity than its predecessor due to reduced amphiphilicity and dipole moment. Princiotto *et al*. modified the retinoid biphenyl core groups allows for the optimization of antimicrobial profiles with minimized cytotoxicity [114]. Compound 17 had a t-butyl oxime on carbon 2 and showed potent antimicrobial activity with low cytotoxic activity. The study also revealed that the presence of two polar groups and a rigid, compact molecular framework may be crucial for facilitating membrane binding and penetration.

Research combined the AlphaFold2 protein structure database with in silico drug screening identified that steroid drugs quinestrol potently inhibited cytochrome bd in both *E. coli* and *S. aureus*, yet only exhibited bactericidal toward *S. aureus*. Ethinylestradiol showed weak inhibition of *E. coli* bd-I, underscoring quinestrol's unique dual antimicrobial profile [115]. This study has revealed that steroids are a promising scaffold for drug development, exhibiting activity against diverse quinone-associated bd-type and heme-c oxidopperases.

Omega-3 PUFAs, including EPA and DHA from fish/spices, demonstrate cardiovascular benefits. DHA-derived Resolvin D1 (RvD1) exhibits potent anti-inflammatory activity in several animal models [116]. This pro-resolving mediator belongs to lipid mediators (lipoxins, resolvins, maresins, protectins) known to mitigate local/systemic inflammation [117]. RvD1 showed good activities against both *S. aureus* and *Candida parapsilosis*, significantly reducing their planktonic growth and biofilm formation. The key findings were that RvD1 reduced biofilm biomass and metabolic activity, induced the production of ROS, downregulated genes related to biofilm formation, and eradicated mature biofilms on silicone surfaces without exhibiting cytotoxicity. These findings highlighted RvD1 as a promising candidate for the treatment of mixed bacterial/fungal infections, potentially overcoming antimicrobial resistance, and necessitated further *in vivo* studies to confirm its therapeutic potential [118].

Furosemide, an FDA-approved diuretic, had the antibacterial sulfonamide moiety and was modified by attaching an aryl group to furosemide at position 1 through various linkers. The compounds 3b and 4a showing particularly strong effects and a low toxicity profile. Furosemide and its analogs showed anti-PBP2a activity which may associated with their antibacterial mechanism [119].

Promethazine, traditionally an antihistamine, exhibited significant antimicrobial activity against gram-positive cocci involved in infectious endocarditis, effectively reducing the resilience of biofilms. Promethazine showed synergistic effects when combined with conventional antibiotics such as vancomycin, oxacillin, and ceftriaxone, particularly against biofilms grown *ex vivo* on porcine heart valves [120].

Levocetirizine dihydrochloride (LVC), a famous antihistaminic drug, was loading into terpene-enriched vesicles (TPs) to augment the antibacterial activity [121]. The results demonstrated that TPs gel loaded with LVC exhibited sustained release, effectively inhibited and eradicated MRSA biofilms, and possessed potent antibacterial activity against MRSA. Computational and *in vivo* studies showed that LVC had favorable binding affinity with the MRSA tyrosyl-tRNA synthetase and TPs gel enhanced the skin permeation of LVC. Histopathological evaluation confirmed the safety of TPs gel.

Otilonium bromide (OB) is a FDA- approved antispasmodic agent for irritable bowel syndrome. It has been demonstrated to inhibit multidrug-resistant *A. baumannii* and *S. aureus* via the bacterial cell membrane destruction and cellular homeostasis disruption [122]. Zhou *et al*. systematically evaluated this drug, OB showed effects against *S. aureus* (MICs: 4-8 μg/ml) and biofilm eradication at 16-64 μg/ml concentrations [123]. In the MASA–infected mouse peritonitis model, it reduced bacterial load and improve mouse survival. Through mechanism research they found OB was a membrane-targeting agent but more research was needed to understand its binding site and molecular mechanism.

Gilbert-Girard *et al*. discovered the multiple sclerosis immunomodulator fingolimod as a potent antibacterial agent, effective against *S. aureus* and drug-resistant *A. baumannii* [124]. It inhibits quorum sensing and showed no resistance development over 20 days. Subsequent structural modifications of fingolimod’s aromatic ring, eightcarbon chain, and polar head group yielded derivatives with enhanced antibacterial properties [125]. Chain elongation and aromatic ring modification improved anti-*S. aureus* activity, while hydrocarbon chain repositioning and hydroxyl group addition extended efficacy to Gram-negative bacteria. The team theorized that other S1PR modulators might offer similar antibacterial benefits and began investigating their potential as repurposed antibacterial agents [126]. They found etrasimod exhibited the most potent inhibitory effects among tested S1PR modulators on *S. aureus*, and also showed activity against other Gram-positive bacteria like *S. epidermidis* and *E. faecalis*. Etrasimod additionally demonstrated synergistic antimicrobial effects with gentamicin.

Antioxidants N-acetyl-L-cysteine (NAC) and cysteamine (Cys) were found effectively eradicated *S. aureus*– *S. pneumoniae* mixed biofilms and individual *S. aureus* biofilms. NAC showed more antibiofilm ability than Cys, it at a concentration of 5 mg/ml killed about 94% of MSSA cells and 99% of MRSA cells and almost eradicated the *S. pneumoniae* population in the mixed biofilms [127].

Avobenzone (AVB), an active ingredient in sunscreens, is a potent Zn-activated inhibitor of MRSA (MICs from 0.62 to 2.5 μM), even at concentrations below the apparent eukaryotic toxicity [128]. Furthermore, AVB-Zn improved mouse survival in a wound model of MRSA infection.

Personal care product preservatives bronopol showed antibiofilm activity against *P. aeruginosa* biofilms (MBEC: 64 mg/l) and *S. aureus* biofilms (MBEC: 256 mg/l), while another preservatives bronidox exhibited MBECs of 128 mg/l [129]. Bronopol and bronidox eradicated *P. aeruginosa* and ruduced about 5 log_10_ biomass in the *S. aureus* population on the mixed-species biofilm. Bronopol and bronidox could synergistically combine with established wound agents (silver nitrate, cetrimide and chlorhexidine) against single-species biofilms of *S. aureus* and *P. aeruginosa*.

PQ401, a diarylurea derivative previously known as an inhibitor of the insulin-like growth factor I receptor, exhibited bactericidal activity and low resistance development, showed synergism with gentamicin, demonstrated efficacy against MRSA in both *C. elegans* and *Galleria mellonella* infection models [130]. PQ401 selectively disrupts bacterial lipid bilayers to kill bacteria. Compared with traditional membrane-active antimicrobial small molecules and peptides, PQ401’s antimicrobial activity was maximized when it existed in its neutral form. This property enables it to bypass the issue of reduced negative charge in AMP-resistant bacteria. Noteworthy, PQ401's therapeutic development requires neutralizing IGF-1R inhibition-induced toxicity (linked to cancers/diabetes) while retaining antimicrobial efficacy.

Rahman *et al*. performed a virtual screening against *S. aureus* FemX and also identified Cystic fibrosis transmembrane conductance regulator (CFTR) modulators lumacaftor had moderate bactericidal activity against *S. aureus* (MIC:128 μg/ml) and inhibited biofilm formation [18, 131].

Hydralazine, antihypertensive agent, demonstrated potent antimicrobial activity against MRSA and MSSA, with MIC and MBC values ranging from 128 to 2,048 μg/ml [132]. It exhibited synergistic effects with oxacillin and vancomycin aganist some isolates, and reduced biofilm viability. Treatment with hydralazine at 2×MIC resulted in significant bacterial cell death, accompanied by DNA damage as confirmed by TUNEL and comet assays. Molecular docking studies revealed hydralazine's affinity for DNA gyrase and TyrRS, key enzymes in bacterial replication.

The antihypertensive agent candesartan cilexetil (CC) exhibited potent antibacterial activity against MRSA with MICs and MBCs ranging from 8–16 μg/ml and 16–32 μg/ml, respectively. CC showed limited cytotoxicity and a low potential to induce drug resistance. Notably, CC exhibited synergistic interactions with gentamicin and tobramycin, significantly enhancing antimicrobial activity against MRSA. Additionally, CC effectively inhibited MRSA biofilm formation at concentrations of 16–64 μg/ml and demonstrated the ability to kill persistent bacteria at 4–8 × MIC. *in vivo* validation in a murine skin abscess infection model further confirmed CC’s therapeutic potential for MRSA-associated infections [133].

Eltabeeb *et al*. reported an nanocomposite alginate hydrogel encapsulating propranolol hydrochloride via ethanol injection, yielding cerosomes with high encapsulation efficiency and stability for MRSA skin infections [134]. The optimized cerosomes exhibited a sustained release *in vitro* and the demonstrated superior antibacterial effects *in vivo* mouse model and biofilm inhibition capabilities compared to traditional solutions.

*Gracilaria edulis* extracts, with various organic solvents, demonstrated potential antibacterial activity against six MDR species namely, *K. pneumoniae*, *P. aeruginosa*, *Salmonella enterica*, MRSA, *Streptococcus pyogenes*, and *Bacillus subtilis*. Following liquid chromatography-mass spectrometry (LC-MS) and gas chromatography-mass spectrometry (GC-MS) analyses, a variety of compounds were identified from these extracts. Through a downstream virtual screening and additional pharmacological screening, eplerenone was highlighted as a potentially potent natural compound that may exert its antibacterial effects by binding to specific proteins in MRSA, such as CapE [135]. Eplerenone is recognized as a potassium-sparing diuretic and has been applied to patients with chronic heart diseases (CHD), and exhibits fewer adverse effects among the spironolactone class of steroidal antimineralocorticoids [136, 137].

3-Acetyl-11-keto-β-boswellic acid (AKBA) is a pentacyclic triterpenoid, is also reported as anti-tumor and anti-inflammatory reagent. AKBA displayed potent antimicrobial activity against MRSA with MICs of 4–8 μg/ml and without inducing resistance after 20 days of continuous passage [138]. AKBA showed synergistic effects when combined with conventional antibiotics such as gentamicin and amikacin. AKBA significantly reduced abscess size and bacterial load *in vivo* studies in murine subcutaneous abscess model without detectable toxicity.

### Antimicrobial Adjuvants

Compounds that lack significant antibacterial properties but augment the potency of antibiotics are referred to as antibiotic adjuvants or potentiators. Antibiotic adjuvants can be classified into three classes based on their mechanisms of action: host-directed therapies, antivirulence drugs and antiresistance drugs.

**Host-directed therapies.** *S. aureus* exploits intracellular survival to infect macrophages, disseminate systemically, evade immune detection, and resist antibiotics (*e.g.*, vancomycin), particularly in hospital-acquired MRSA. Novel strategies complementing traditional antibiotics are critical. Intracellular pathogens subvert host pathways for survival, highlighting the importance of studying host-pathogen interactions to identify therapeutic targets. Host-directed approaches may: (i) block pathogen-hijacked pathways, (ii) enhance antimicrobial immunity, (iii) suppress hyperinflammation, and (iv) stabilize dysregulated host responses. These strategies encompass monoclonal antibodies, cytokines, repurposed drugs, and cellular therapies, offering multifaceted solutions to combat multidrug-resistant infections by targeting both pathogen virulence and host vulnerability [139-141].

DI-87, a host-directed therapy drug, by inhibiting the host cell's deoxycytidine kinase, effectively reduced the apoptosis of phagocytes, enhanced the infiltration of macrophages into the abscesses, and thus decreased the formation of abscesses and bacterial load [142].

Sperry *et al*. combined computational transcriptomics and the Xenopus laevis embryo model to discover and validate drugs that enhance host tolerance to bacterial infections [143]. It founds that Xenopus embryos exhibited natural tolerance to multiple bacterial infections and defined a 20-gene signature for Gram-negative tolerance. Computational analyses revealed conserved pathways, including metal ion homeostasis and hypoxia-inducible factor (HIF-1α) signaling, critical for tolerance. FDA-approved drugs targeting these pathways were repurposed: deferoxamine, an iron chelator, disrupts bacterial iron acquisition (essential for *S. aureus* virulence factors like biofilm formation) while mitigating host oxidative stress; 1,4-DPCA, a HIF-1α agonist, stabilizes HIF-1α to enhance metabolic adaptation (*e.g.*, glycolysis) and suppress hyperinflammation, reducing tissue damage. Although direct validation against *S. aureus* was limited, these drugs improved survival in Gram-negative infections (*e.g.*, Aeromonas hydrophila) without reducing pathogen burden, highlighting their role in tolerance induction. Cross-species transcriptomic comparisons confirmed conservation of metal-binding and hypoxia-response genes, supporting broader applicability. This host-directed approach circumvents antibiotic resistance by enhancing resilience, offering a complementary strategy for *S. aureus* infections. Further studies are warranted to optimize dosing and validate efficacy in gram-positive models.

Substance P (SP), a widely distributed neuropeptide in the nervous and immune systems, significantly augmented the inflammatory and pro-osteoclastogenic responses of murine osteoclasts and osteoblasts to *S. aureus* infection through its interaction with the neurokinin-1 receptor (NK-1R). SP enhanced the production of inflammatory cytokines and pro-osteoclastogenic factors by these bone cells, leading to increased bone resorption and inflammation. Notably, NK-1R antagonists, particularly aprepitant, demonstrated potential in inhibiting SP-mediated actions, mitigating inflammation, and reducing bone loss, suggesting they may be effective therapeutic agents for the treatment of *S. aureus* bone infections [144].

At clinically relevant concentrations, poly (ADP-ribose) polymerase (PARP) inhibitors effectively protected human peripheral blood mononuclear cells from oxidative stress-induced damage [145]. Specifically, olaparib suppressed PARP1 activation, counteracted H2O2-induced depletion of NAD+ and ATP, and prevented mitochondrial membrane depolarization. Furthermore, olaparib significantly reduced the release of inflammatory cytokines (TNF-α, MIP-1α, and IL-10) following LPS stimulation while preserving monocyte and neutrophil functions, including ROS and nitric oxide (NO) production, as well as phagocytic capacity and antimicrobial activity.

**Antivirulence drugs.** *S. aureus* produces diverse virulence factors, including pore-forming hemolysins (*e.g.*, α-toxin), leukotoxins (*e.g.*, PVL), exfoliative toxins, and enterotoxins, which disrupt host cells and immune responses. Toxin production is tightly regulated by quorum-sensing systems, primarily the agr locus, which controls RNAIII-mediated expression of toxins, and other regulators like sae and sarA [146, 147]. Anti-virulence strategies include direct toxin neutralization and indirect approaches targeting regulatory pathways. These strategies aim to attenuate pathogenicity without inducing bacterial lethality, offering alternatives to traditional antibiotics against multidrug-resistant infections [147].

Two repurposed drugs, ticagrelor and oseltamivir, could block the *S. aureus* alpha-toxin's damage to platelets, maintain platelet counts, and thereby enhance the host's defense against *S. aureus* bacteremia [148]. The alpha-toxin activated platelet endogenous sialidase activity, leading to accelerated clearance of platelets through the hepatic Ashwell-Morell receptor, and that ticagrelor and oseltamivir were able to inhibit this process, reduce thrombocytopenia, and improve survival rates in a murine model of *S. aureus* infection. The highlight of this research was the discovery of platelets' direct bactericidal function against *S. aureus* bacteremia and the novel strategy of enhancing host defense by pharmacologically intervening in the platelet clearance mechanism.

Fenoprofen, a NSAID, specifically targets the SaeR protein, a crucial component of *S. aureus*'s SaeRS two-component system, which governs virulence factor expression and biofilm formation [17]. By inhibiting SaeR's transcriptional regulation, Fenoprofen reduces virulence gene expression without hampering bacterial growth. It combats *S. aureus* virulence through multiple pathways: inhibiting biofilm formation, modifying biofilm structure to increase susceptibility to host immune attacks, and diminishing bacterial invasion into host cells while enhancing infection clearance. *in vitro* and *in vivo* experiments reveal that fenoprofen significantly lightens bacterial burden, mitigates osteolysis, and restores walking ability in mice with implant-associated infections.

FDA-approved NSAID Diflunisal had the potential as an osteoprotective, antivirulence therapy for *S. aureus* osteomyelitis [149]. Diflunisal potently inhibited bacterial cytotoxicity towards osteoblasts by limiting alpha-type phenol soluble modulins (key contributors to the pathogenesis of *Staphylococcal osteomyelitis*) production via inactive agr locus. *In vivo* application of diflunisal via a drug-eluting, bioresorbable foam effectively inhibited bone destruction during *S. aureus* osteomyelitis. Notably, diflunisal-mediated Agr suppression may paradoxically promote biofilm proliferation in *S. aureus* [150, 151], warranting combination with conventional antibiotics and restriction to acute osteomyelitis rather than chronic/implant-related infections.

Bumetanide, a diuretic, targeted the AgrA quorum sensing regulator in *S. aureus*, resulting in significant antivirulence effects [152]. At 0.1 μM, bumetanide outperformed the reference compound diflunisal, achieving 70% inhibition. It downregulated virulence genes, including RNAIII, Hla, PSMα, and lukS-PV, highlighting its potential. *in vivo*, bumetanide controlled infection and aided wound healing in a dermonecrosis mouse model without inducing tolerance or resistance. Its lack of dermal toxicity positions bumetanide as a candidate for topical treatments of skin infections.

**Antiresistance drugs.** Antiresistance drugs directly counter bacterial defense mechanisms through enzymatic inhibition or genetic interference with resistance determinants. This category encompasses β-lactamase inhibitors, efflux pump blockers, plasmid conjugation disruptors and so on. By neutralizing resistance genes/proteins, these adjuvants restore the therapeutic activity of legacy antibiotics against multidrug-resistant strains, effectively expanding the clinical utility of existing antimicrobial arsenals.

Lignin restores β-lactam efficacy against resistant *S. aureus* by disrupting cell envelope homeostasis. It compromises membrane integrity, modifies peptidoglycan structure, and induces stress responses through pH fluctuations and altered carotenoid synthesis [153]. Transcriptomics shows upregulation of envelope stress regulators (σB/VraRS), lipid metabolism, and resistance genes, alongside downregulation of cell wall biosynthesis pathways. Lignin pretreatment reduces detergent tolerance and enhances β-lactam activity against genetically resistant strains, while exhibiting no cytotoxicity in human keratinocytes at effective concentrations. These coordinated effects suggest lignin's dual role as both a membrane-active compound and β-lactam adjuvant.

Hypericin (HYP), a bioactive naphthodianthrone constituent of St. John's wort, was identified as a novel sarA inhibitor that potentiates β-lactam antibiotic efficacy against MRSA. Mechanistic studies revealed HYP-mediated downregulation of agr RNAIII transcript levels and concomitant suppression of its downstream virulence determinants [154]. Furthermore, systematic investigations employing both *in vitro* susceptibility testing and an experimental endocarditis model established that sarA modulates β-lactam resistance phenotypes in MRSA through transcriptional regulation of mecA expression [155]. HYP reduced β-lactam antibiotic MICs by 2- to 64-fold, weakened MRSA's biofilm formation and fibronectin adhesion, and improved treatment in a murine bacteremia model.

Chang *et al*.’s study explored three comprehensive strategies to identify novel antibacterials and adjuvants via computational and multidisciplinary experimental methods [156]. First, non-β-lactam inhibitors targeting penicillin-binding proteins (PBPs)—specifically oxadiazoles and quinazolinones—exhibited potent activity against MRSA and other Gram-positive pathogens. Second, mechanistic characterization of allosteric regulation in *S. aureus* PBP2a revealed that ligands such as ceftaroline bind 60 Å distal to the catalytic site, inducing conformational activation and synergistic β-lactam potentiation. Third, novel adjuvants were identified: triarylimidazoles and cinnamonitrile analogs reduced oxacillin MICs in MRSA by inhibiting BlaR1 phosphorylation, while bulgecin A disrupted lytic transglycosylases (*e.g.*, *P. aeruginosa* Slt), synergizing with β-lactams to induce cell-wall lysis.

Dey *et al*. successfully designed and synthesized a small molecular adjuvant known as small molecular adjuvant (SMA) by tuning structural parameters, such as the balance between hydrophilic and hydrophobic groups, the spatial positioning of hydrophobicity, and hydrogen bonding interactions, which led to the perturbation of bacterial cell membranes and the inhibition of bacterial efflux pumps [157]. SMA significantly enhanced the efficacy of various traditional antibiotics against drug-resistant Gram-negative bacteria (32- to 512-fold). It also demonstrated good biocompatibility and antimicrobial efficacy in both *in vitro* and *in vivo* models. Furthermore, the combination therapy of SMA with fusidic acid also exhibited antimicrobial activity against Gram-positive bacteria, particularly MRSA.

Six FDA-approved drugs (raloxifene, ezetimibe, propafenone, nefazodone, chlorprothixene, pyrvinium) exhibited NorA efflux pump inhibitory activity through integrated virtual screening and *in vitro* validation [158]. These drugs significantly suppressed norfloxacin and ciprofloxacin efflux in *S. aureus*, thereby increasing bacterial susceptibility to fluoroquinolones. Notably, pyrvinium combined with ciprofloxacin achieved substantial eradication of preformed biofilms and reduced bacterial load in murine thigh infection models.

The combination of DNase I and alginate lyase with broad-spectrum antibiotics (meropenem, tobramycin) effectively disrupted dual-species *S. aureus*-*P. aeruginosa* biofilms, resulting in marked biomass reduction and bacterial clearance. The synergistic action was most pronounced in highly viscoelastic biofilm architectures [159].

## Challenges and Future Perspectives in Drug Repurposing

Antibacterial drug repurposing presents a strategic approach to address antimicrobial resistance, though significant challenges persist. Mechanistic uncertainties remain paramount: numerous candidates lack validated molecular targets or demonstrate insufficient penetration against biofilms and Gram-negative pathogens due to impermeable outer membranes and efflux systems [13]. Translational barriers emerge from interspecies variations in pharmacokinetics and immune-microbial interactions, diminishing the predictive capacity of preclinical models [160]. Toxicity assessment introduces additional challenges, particularly for drugs initially developed for chronic conditions, where antimicrobial effectiveness may require doses beyond established safety parameters [161]. Regulatory and economic obstacles hinder advancement; substantial costs for clinical validation of new indications, combined with minimal commercial incentives for narrow-spectrum agents, inhibit investment [162]. Limited collaboration between academia and industry compounds inefficiencies in data sharing and resource allocation, while global disparities in diagnostic infrastructure limit the deployment of pathogen-specific therapies [163].

We remain optimistic about the potential of drug repurposing. Developments in AI-integrated multi-omics platforms and structural biology are expediting target identification and rational drug optimization [13, 164]. Regulatory bodies are investigating adaptive pathways to facilitate approvals for high-need infections, bolstered by public-private partnerships like CARB-X and the MRC-AstraZeneca initiative, which promote open-access compound libraries and risk-sharing funding models [13, 165]. Investments in point-of-care diagnostics and antimicrobial stewardship programs are improving precision in drug deployment [163]. The increasing political acknowledgment of antimicrobial resistance, demonstrated through global funding initiatives, highlights a dedication to coordinating policy and innovation [13, 160]. By harmonizing scientific rigor, economic incentives, and equitable access frameworks, drug repurposing is poised to deliver clinically impactful antimicrobials, offering a pragmatic complement to novel drug discovery in the global fight against antimicrobial resistance.

## Supplemental Materials

Supplementary data for this paper are available on-line only at http://jmb.or.kr.
